# Structural effects of clinically observed mutations in JAK2 exons 13-15: comparison with V617F and exon 12 mutations

**DOI:** 10.1186/1472-6807-9-58

**Published:** 2009-09-10

**Authors:** Tai-Sung Lee, Wanlong Ma, Xi Zhang, Hagop Kantarjian, Maher Albitar

**Affiliations:** 1 Biomedical Informatics and Computational Biology, and Department of Chemistry, University of Minnesota, 207 Pleasant Street, S.E., Minneapolis, MN 55455, USA; 2 Quest Diagnostics Nichols Institute, San Juan Capistrano, California, USA; 3 Department of Leukemia, University of Texas, MD Anderson Cancer Center, Houston, Texas, USA

## Abstract

**Background:**

The functional relevance of many of the recently detected JAK2 mutations, except V617F and exon 12 mutants, in patients with chronic myeloproliferative neoplasia (MPN) has been significantly overlooked. To explore atomic-level explanations of the possible mutational effects from those overlooked mutants, we performed a set of molecular dynamics simulations on clinically observed mutants, including newly discovered mutations (K539L, R564L, L579F, H587N, S591L, H606Q, V617I, V617F, C618R, L624P, whole exon 14-deletion) and control mutants (V617C, V617Y, K603Q/N667K).

**Results:**

Simulation results are consistent with all currently available clinical/experimental evidence. The simulation-derived putative interface, not possibly obtained from static models, between the kinase (JH1) and pseudokinase (JH2) domains of JAK2 provides a platform able to explain the mutational effect for all mutants, including presumably benign control mutants, at the atomic level.

**Conclusion:**

The results and analysis provide structural bases for mutational mechanisms of JAK2, may advance the understanding of JAK2 auto-regulation, and have the potential to lead to therapeutic approaches. Together with recent mutation profiling results demonstrating the breadth of clinically observed JAK2 mutations, our findings suggest that molecular testing/diagnostics of JAK2 should extend beyond V617F and exon 12 mutations, and perhaps should encompass most of the pseudo-kinase domain-coding region.

## Background

Protein kinases play critical key roles in cellular signal transduction and can be activated by either phosphorylation or interaction with other proteins or ligands [1-4]. However, surprisingly little is known about protein-protein interactions involved in kinase activation processes. Scaffolds have been shown as allosteric regulators of kinases; symmetrical dimers of kinases could be involved in auto-phosphorylation regulation; and many protein kinases may be regulated via N-lobe interactions. In many protein kinases, activity is regulated by the coupling between the C-helix and regulatory domains [5-7]. In others, the catalytic domains act as their own regulatory regions [8,9]. Recently, the MAP2K MEK6 was demonstrated to be auto-regulated via the formation of a symmetrical dimer [10].

Janus kinase 2 (JAK2) is a protein tyrosine kinase that transduces cellular signals through the JAK-STAT pathways [11,12]. Deregulation of JAK2 is thought to be associated with hematopoietic disorders and oncogenesis [13-17], especially in patients with *BCR-ABL*-negative myeloproliferative neoplasms (MPNs) [18]. However, the regulation mechanism of JAK2 remains elusive. Its kinase domain, JH1, is likely negatively regulated [19,20] by the adjacent pseudo-kinase domain, JH2, probably in a manner similar to the mechanism of JAK3 regulation [21]. Although other kinase/pseudo-kinase systems have been reported recently [22], the detailed interactions of JH1/JH2 are not well-understood, owing to a lack of available experimental structures. A theoretical structure model of the whole JAK2 assembly has been built using homology modeling methods and co-evolution data [23,24]. In this model, the JH1 and JH2 domains face each other in an anti-symmetrical way; that is, the JH1 N-lobe contacts the JH2 C-lobe while the JH1 C-lobe contacts the JH2 N-lobe, with a helix-helix contact between the JH1 C-helix and the JH2 C-helix. This anti-symmetrical interface has not been reported in any other kinase/pseudo-kinase pair systems.

In 2005, studies identified the V617F mutation of the JAK2 JH2 domain in large numbers of patients with diverse clonal myeloid disorders [14,17,25-33]. The surprising frequency of this mutation suggests that V617 may play important roles in the auto-regulation of JAK2 and could become an important molecular target for new drug design. The V617F findings also imply that other mutations with similar phenotypes might have been ignored in the past; Ma *et al *[34] recent confirmed this idea through clinical evidence documenting mutations in exons 12 through 15 in patients with MPN-related phenotypes.

It has been suggested that the theoretical model outlined above may be able to explain the mutational effect of JAK2 V617F at the atomic level [35]. Starting from this theoretical model, a homology-based structure has been used to explain the mutational effect of an exon-12 mutant [36]. Homology modelling technique has also been applied to predict the mutational effect of an exon 12 deletion [36]. Although those models demonstrate the relative position of V617 to the JH1/JH2 domain, further atomic level details, not seen in those models, are desperately needed to explain the constitutive activation caused by V617F.

**Figure 1:**
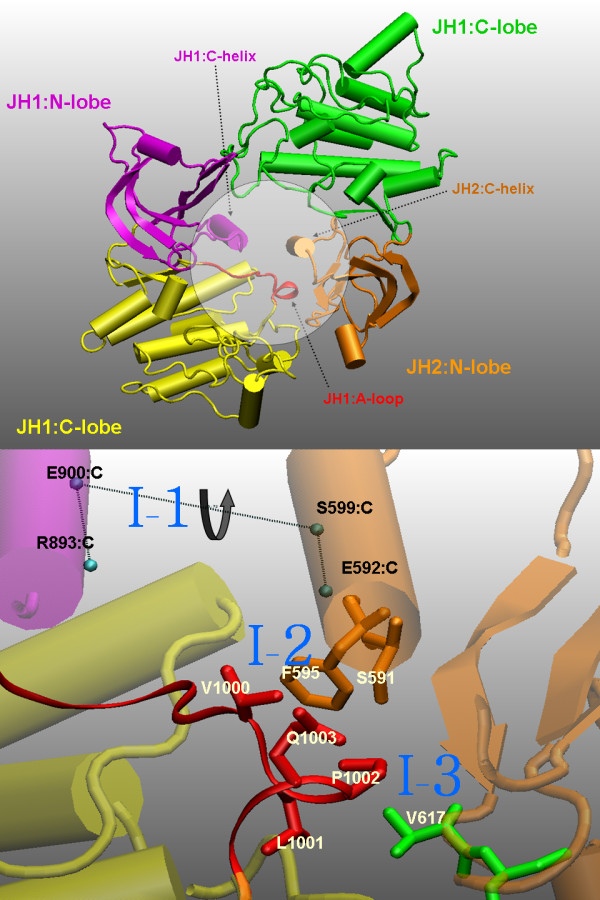
**The simulation-derived JH1/JH2 interface of JAK2**. ***Upper***: The overall relative positions of JH1 and JH2. JH1 and JH2 form an anti-symmetrical pair (dimer). The N- and C- lobes of JH1 and JH2 are shown in different colors and are indicated by text with the corresponding color. The circled area is the main region of the JH1/JH2 interface relevant to JAK2 auto-regulation. The red loop is part of the JH1 activation loop (residues 995 to 1005). ***Lower***: A more detailed view of the JH1/JH2 interface. The curved arrow indicates the torsion angle between the C-helices of JH1 and JH2. The blue labels (I-1, I-2, and I-3) indicate the sub-interfaces, or groups of interactions, defined in the previous work[42]: I-1, interactions between the C-helices of JH1 and JH2; I-2, interactions between the JH1 activation loop and the JH2 C-helix; and I-3, interactions between the JH1 activation loop and residues near position 617 of JH2.

Molecular dynamics simulations of biomolecules have provided useful information complementary to experimental evidence [37-39]. We recently reported 60-ns molecular dynamics simulations of the wild-type and V617F mutant JAK2 proteins, starting with the theoretical model mentioned above [23,40]. Although the overall fold is maintained, the local conformations obtained from our simulations are significantly different from the starting theoretical homology model. More importantly, the simulation results now are able to explain the auto-regulation of JAK2 and the constitutive activation due to V617F in detail. There are three key groups of interactions between the JH1 and JH2 domains, holding JH1 in a closed inactive conformation. In the V617F mutant, F617 interacts with F595 and blocks other key interactions, resulting in loss of the JH1/JH2 interactions and forcing JH1 back to it open, active conformation (Figure 1).

**Figure 2:**
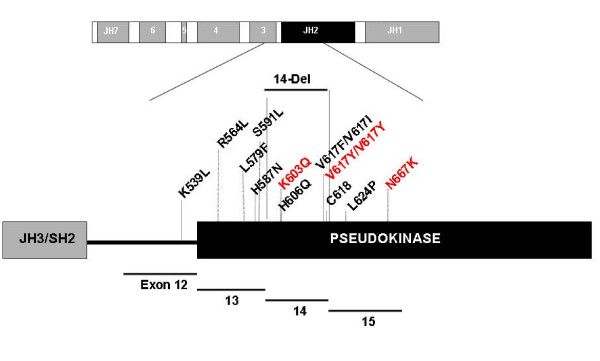
**Schematic diagram of JAK2 gene and protein showing the homology domains (JH1-7)**. The schematic protein shows the distribution of the studied mutations along with the corresponding exons. Mutations depicted in red are control mutations and mutations depicted in black have been reported in patients with MPN.

Nevertheless, only the wild-type and one mutant (V617F) JAK2 were simulated in our previous study, hence the prediction of the JH1/JH2 domain interactions is not yet conclusive. Here we extend the simulations to newly discovered, clinically observed mutants, along with a set of presumably benign control mutants (Figure 2). Our findings here unveil the JAK2 auto-regulation mechanism in much greater detail, and simulation results provide a plausible explanation for the effects of each mutant at the atomic level. This study confirms the anti-symmetrical JH1/JH2 interface and, to our best knowledge, is the first to reconcile extensive clinically observed mutants through computational simulations of JAK2. These findings may provide the framework for rational drug design targeting various JAK2 mutants.

## Results and discussion

### JH1/JH2 Interface and Mutant Selection

The JH1/JH2 interface derived from our previous simulation work is summarized in Figure 1. Three key groups of interactions are shown: I-1 is the helix-helix interactions between the JH1 C-helix and the JH2 C-helix; I-2 is the interactions between the JH1 activation loop (A-loop) and the JH2 C-helix; and I-3 is the interactions between the activation loop of JH1 and a region of contact in JH2, including V617.

Ma *et al *[34] recently reported detection of several mutations in exons 12 to 15, based on analysis of approximately 20,000 clinical blood samples. Their findings suggested that molecular testing of *JAK2 *mutations should not be restricted to the V617F and exon 12 mutations, but perhaps should extend to most of the JH2 pseudo-kinase domain. Hence, the mutants used in our simulations were selected from the report of Ma et al (Figure 2): K539L in exon 12; R564L, L579F, H587N, and S591L in exon 13; 14-del (deletion of entire exon 14, corresponding to residues 592 to 622), H606Q, V617I, V617F, and C618R in exon 14, and L624P in exon 15. All were detected in patients with MPN. In addition, control simulations were performed using the K603Q/N667K double-mutant, V617C, and V617Y. The double-mutant should be benign because Q603/K667 is found in the JAK2 of other mammals, including pig, chicken, mouse, and rat; only human and pony show the "un-mutated" sequences (K603/N667). V617C and V617Y have never been reported in patients with MPN, despite the prevalence of V617F and experimental data demonstrating that V617C is benign [41].

### Distortions of JH1/JH2 Interactions Due to Various JAK2 Mutations

Long time (60 ns each) full-scale molecular dynamics simulations were performed for each mutant, using simulation protocols described previously [42] and in the Methods section. The simulations performed here require at least 20 ns to reach steady-state, and the resulting mutational structure changes cannot be obtained by simple homology modelling methods [36].

**Figure 3:**
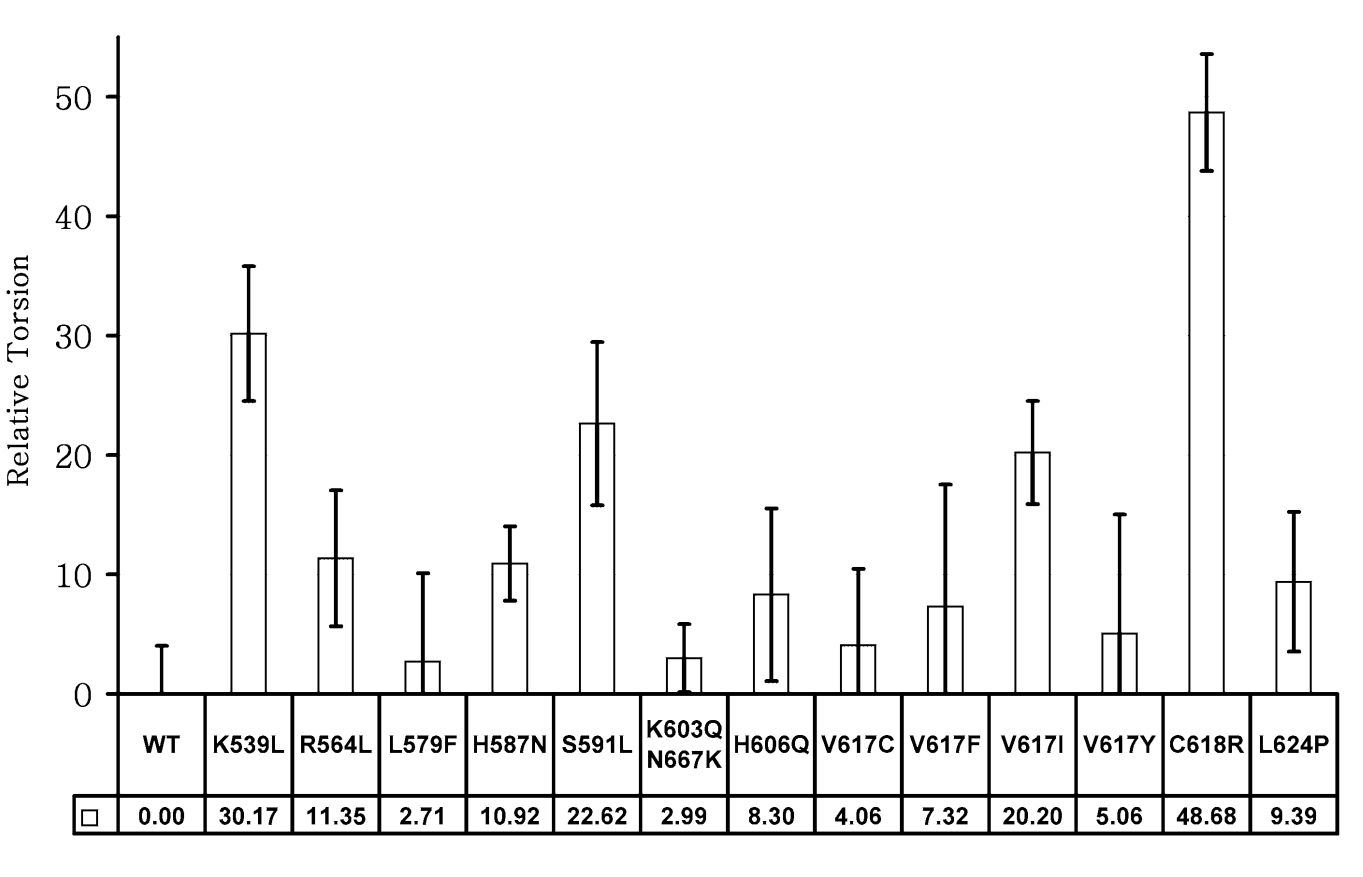
**The relative torsion angle between the JH1 C-helix and the JH2 C-helix**. This angle is defined as the dihedral angle between the carbonyl carbon atoms of residues R893, E900, S599, and E592. Data across the bottom of the figure indicate the difference in torsion of each mutant relative to the average torsion of the wild-type JAK2 simulation (-5.71 degrees). Mutations with larger relative torsion angles exhibit greater deviation from wild-type JAK2 and are thus more likely to be deleterious. The error bars are the standard deviations of the calculated values from the last 30-ns trajectory for each mutant. Entries were obtained with a sampling frequency of 10 ps; i.e., 3,000 sample data points for each mutant simulation.

The use of various distances between JH1 and JH2 proved useful in characterizing the JH1/JH2 interface in our previous work [42]. However, the large number of distances needed to monitor the JH1/JH2 interface makes a succinct summary of the results difficult. Hence, instead of inter-atomic distances, we used the torsion angle between the C-helices of JH1 and JH2 to monitor the I-1 group of interactions. Figure 3 shows the relative torsion angle, defined as the dihedral angle between the carbonyl carbon atoms of residues R893, E900, S599, and E592, of all mutants from simulations,. I-1 interactions are severely changed in four mutants: K539L, S591L, V617I, and C618R. The data for 14-del is not shown since this mutant lacks the entire JH2 C-helix. I-1 interactions are more-or-less maintained in four mutants: L579F, K603N667K, V617C, and V617Y.

**Figure 4:**
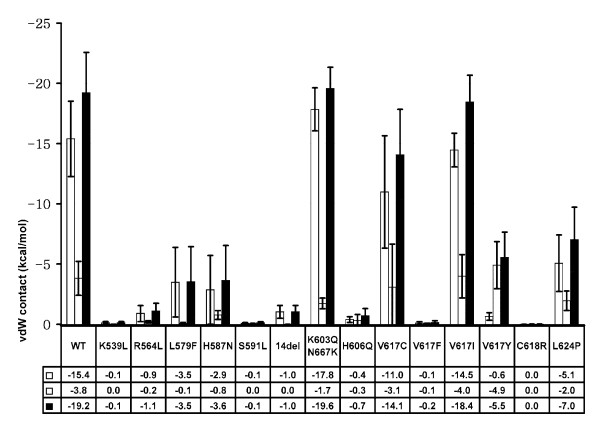
**van der Waals contact energies between different groups for each JAK2 mutant**. Three sets of van der Waals contact energies were calculated (also see the main text): vdw(JH1:A, JH2:C) is the van der Waals contact between the JH1 activation-loop (residues 995 to 1005) and the JH2 C-helix (resides 585 to 595), colored white; vdw(JH1:A, JH2:V) is the van der Waals contact between the JH1 activation-loop and JH2 residues 614 to 619, colored light gray; and vdw(JH1:A, JH2:CV) represents the summation of the above two terms, colored dark gray. A larger (magnitude) van der Waals contact indicates a stronger interface. Wild-type JAK2 (WT) has the strongest interactions whereas a deleterious mutant, such as 14-del, will have very weak interactions. The error bars indicate the standard deviations of the calculated values from the last 30-ns trajectory for each mutant. Entries were obtained with a sampling frequency of 10 ps (i.e., 3,000 sample data points for each mutant simulation).

For I-2 and I-3 the van der Waals contact, a measure of inter-atomic interaction, was used to quantitatively analyze the interactions. The following van der Waals contacts were measured:

• ***vdw(JH1:A, JH2:C)***: the van der Waals contact between the JH1 activation-loop (residues 995 to 1005) and the JH2 C-helix (resides 585 to 595). This term mainly measures the I-2 interactions.

• ***vdw(JH1:A, JH2:V)***: the van der Waals contact between JH1 activation-loop and JH2 residues 614 to 619. This term mainly measures the I-3 interactions.

• ***vdw(JH1:A, JH2:CV)***: the summation of the above two terms, vdw(JH1:A, JH2:C) and vdw (JH1:A, JH2:V). This term measures the total interactions of I-2 and I-3.

van der Waals contacts usually have negative values, indicating the molecular attracting force between atoms in molecules. A van der Waals contact larger in magnitude (with a negative sign) indicates that the two groups of measured atoms are closer; the van der Waals contact will be near 0 when two groups of measured atoms are far away from each other. Hence, the van der Waals contacts in Figure 4 can be thought of as the measure of the closeness between the JH1 activation loop and the JH2 domain (I-2 and I-3 interactions shown in Figure 1 lower panel). The total term, *vdw(JH1:A, JH2:CV)*, indicates how much the JH1 activation loop is "locked" by the JH2 domain. When the contact is strong, there is no space for ATP or ligands to enter the active site and the JH1 kinase domain is thus locked and inactive. The results suggest that wild-type, K603Q/N667K, V617C, and V617I JAK2 have significantly larger contacts between the JH1 activation loop and the JH1 domain, while the K539L, R564L, S591L, 14-del, H606Q, V617F, and C618R mutants have very small contacts.

Table 1 summarizes the mutational effect annotations from simulations and confirms that the control mutants K603Q/N667K and V617C are likely to be benign, as well as the comparison with experimental/clinical evidence. This result is consistent with the fact that all mutants except K603Q/N667K, V617C, and V617Y correspond to MPN-positive cases. V617C in fact has been shown to be benign experimentally [41]. In addition, the simulation results of V617F and K539L suggest that both are deleterious mutants and cause significant changes in the JH1/JH2 interface, well in line with clinical data showing that both are transforming [17,43,44]. Hence V617F and K539L can be considered as the positive control simulations. Detailed analyses and the origins of the mutation effects for each mutant will be discussed in the following sections.

**Table 1:** Summary of predicted effects of JAK2 mutations.

**Mutation**	**Interactions**			**JH1/JH2 interface Openness**	**MPD implication**[17,34,43,44]	**Experimental evidence**[41]
	**I-1**	**I-2**	**I-3**			
**Wild-Type**	O	O	O	O	no	benign
**K539L**	XX	XX	X	XX	yes	
**R564L**	X	XX	X	XX	yes	
**L579F**	O	X	X	X	yes	
**H587N**	X	X	X	X	yes	
**S591L**	XX	XX	X	XX	yes	
**14-del**	XX	XX	X	XX	yes	
**K603QN667K**	O	O	O	O	no	
**H606Q**	X	XX	X	XX	yes	
**V617C**	O	O	O	O		benign
**V617F**	X	XX	X	XX	yes	deleterious
**V617I**	XX	O	O	XX		deleterious
**V617Y**	O	X	O	X		
**C618R**	XX	XX	X	XX	yes	
**L624P**	X	X	O	X	yes	

Summary of predicted effects of JAK2 mutations on I-1, I-2, and I-3 interactions and total effects on the JH1/JH2 interface; and the comparison with experimental/clinical evidence. "O" indicates benign; "X", mildly deleterious; "XX", severely deleterious. The "JH1/JH2 openness" is defined as severely deleterious when I-1 or I-2 is severely deleterious.

## Origins of mutational effects

### V617X Mutants

**Figure 5:**
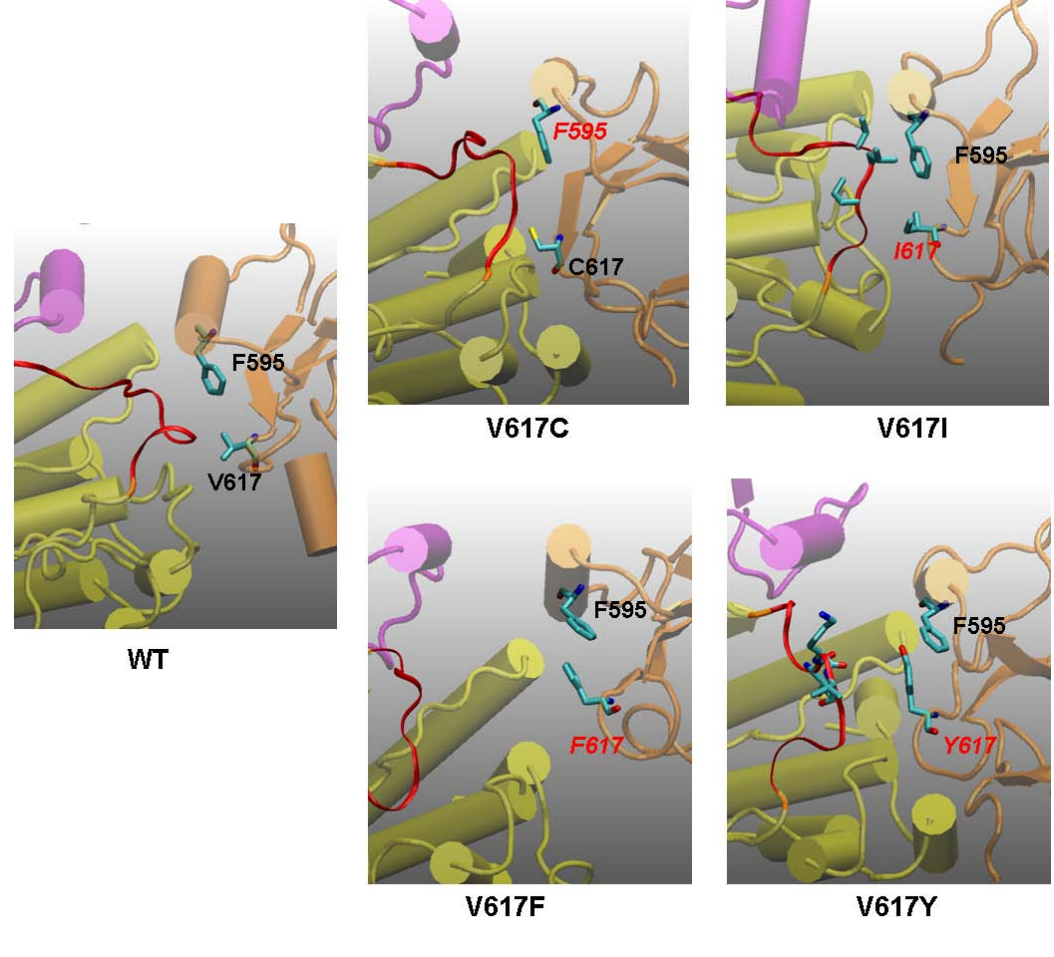
**Possible origins of V617X mutational effects derived from the snapshots of various mutant simulations at 44 ns**. Color codes are the same as in Figure 1. *In wild-type JAK2 (WT)*, the JH1 activation loop is in contact with V617 and nearby residues, as well as the residues of the JH2 C-helix (especially F595 and S591). *V617C *is very similar to WT. All key interactions seem to be kept. However, C617 binds to different parts of the JH1 activation loop: it is now close to K1005 rather than L1001 and P1002. K1005 is the edge of the red region shown in the figure. *V617I *is similar to V617C and WT in that I-2 and I-3 are maintained (i.e., the JH1 activation loop is locked by the JH2 C-helix and residues in the V617 region). However, the bulky side chain of I617 pushes the JH1 activation loop toward the I-1 interface (JH1 C-helix/JH2 C-helix) and breaks the I-1 interactions. ***In V617F***, F617 interacts with F595 and hence blocks the contacts between the JH1 activation loop and the JH2 C-helix. The JH1 activation loop is wide-open and highly accessible. ***In V617Y***, Y617 also interacts with F595 and blocks the contacts between the JH1 activation loop and the JH2 C-helix. However, the polar tail of Y617 moves near the interface between the JH1 C-helix and the JH2 C-helix, forming multiple hydrogen-bonds with nearby water molecules and the JH1 activation loop (not shown). The result is that the JH1/JH2 interface is not as open as in the case of V617F.

Figure 5 shows representative snapshots for mutations at the 617 position. In wild-type JAK2, the JH1 activation loop is in contact with V617 and nearby residues, as well as the residues of JH2 C-helix, especially F595 and S591. I-1, I-2, and I-3 interactions are well preserved (Figure 4 and Figure 5). In V617F, F617 interacts with F595 and hence blocks the contacts between the JH1 activation loop and the JH2 C-helix. The JH1 activation loop is wide-open and highly accessible. I-1, I-2, and I-3 interactions are both lost (Figure 4 and Figure 5). This mutation has been discussed in detail in our previous study [42].

V617C is very similar to wild-type and all three sets of interactions (I-1, I-2, and I-3) are well preserved. V617C is thus a benign mutation, consistent with a recent experimental study [41]. However, close examination of the local conformation indicates that C617 binds to the JH1 activation loop in a slightly different way. In WT, V617 mainly contacts with hydrophobic residues V1000, L1001, and P1002, while in V617C, C617 slowly moves to form polar interactions with residues 1003 to 1005 after 40 ns. This implies that while V617C does not cause constitutive activation, it may induce different phosphorylation patterns at the JH1 activation loop.

V617I is similar to wild-type in that I-2 and I-3 are maintained; that is, the JH1 activation loop is locked by the JH2 C-helix and V617 region residues. However, the bulky side chain of I617 pushes the JH1 activation loop toward the I-1 interface (JH1 C-helix/JH2 C-helix) and breaks the I-1 interactions. This may imply that a mutation with a large non-polar side-chain will yield similar results.

In V617Y (Figure 4 and Figure 5), Y617 also interacts with F595 and blocks the contacts between the JH1 activation loop and JH2 C-helix in a way very similar to V617F, in that the I-2 interactions (vdw [JH1:A, JH2:C]) are severely damaged. However, while its aromatic ring interacts with F595, the polar tail of Y617 moves near the interface between the JH1 C-helix and the JH2 C-helix, forming multiple hydrogen-bonds with nearby water molecules and with the JH1 activation loop (not shown), indicated by the small decrease in the I-3 interactions (vdw [JH1:A, JH2:V]). The result is that the JH1/JH2 interface is not as open as with F617. Based on the results of V617F and V617Y, V617W would have mutational effects similar to those of V617F, since it will also interact with F595 and does not have a polar tail like V617Y. This prediction is in fact consistent with recent observations [41].

The simulation results of V617 mutants demonstrate the important roles that residue 617 side chain plays in JH1/JH2 interactions. An aromatic ring side chain (V617F,Y) interacts with F595 to block the JH1 activation contact; a polar side chain (V617C) could change the local conformation and form a contact with K1005; and a large hydrophobic side chain (V617I) could interfere with the I-1 interactions.

### S591

**Figure 6:**
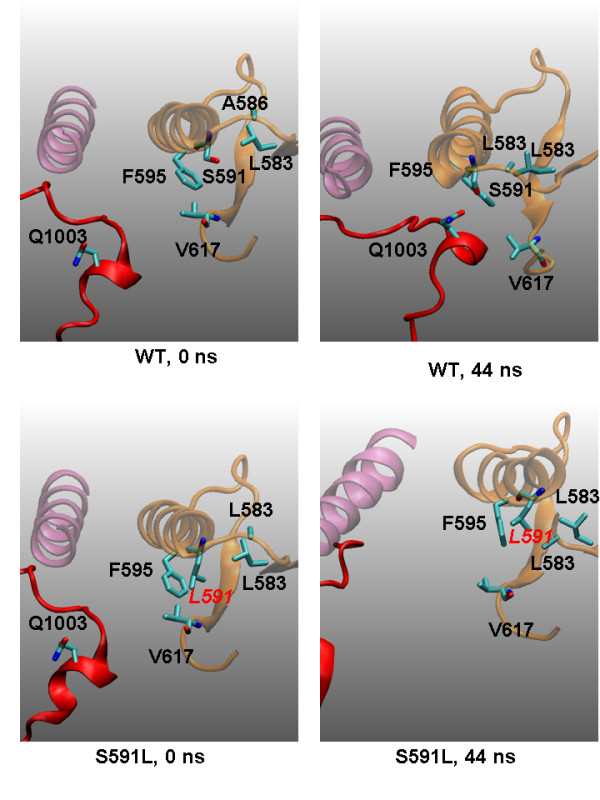
**Possible origins of S591L mutational effects derived from the snapshots of various mutant simulations at 0 and 44 ns**. Color codes are the same as in **Figure 1**. At the very beginning of simulations (0 ns), S591L has the same conformation as wild-type JAK2 (WT). At 44 ns, the JH1/JH2 interface is well formed in WT while L591 forms hydrophobic contacts with nearby F595, L583, and A586, totally disrupting the JH1/JH2 interface.

In our previous study, we predicted that a mutation of S591 would break the polar interaction between S591 and Q1003 and hence affect the I-1 interactions. S591L was indeed observed in MPN patients recently [34]. As shown in Figure 6, S591L has the same conformation as wild-type JAK2 at the very beginning of simulations (0 ns). At 44 ns, the JH1/JH2 interface is well formed in wild-type JAK2; in S591L JAK2, L591 forms hydrophobic contacts with nearby F595, L583, and A586. The I-1 interface is totally distorted, and the I-2 and I-3 falls apart as well. The result suggests that S591L will be a severely deleterious mutation and will cause constitutive activation of JAK2. The mechanism may reflect the hydrophobic nature of the L591 residue in that its side chain is repelled by the JH1/JH2 interface region, which is highly solvent-accessible and is surrounded with many water molecules.

### K539L, C618R, and R564L

**Figure 7:**
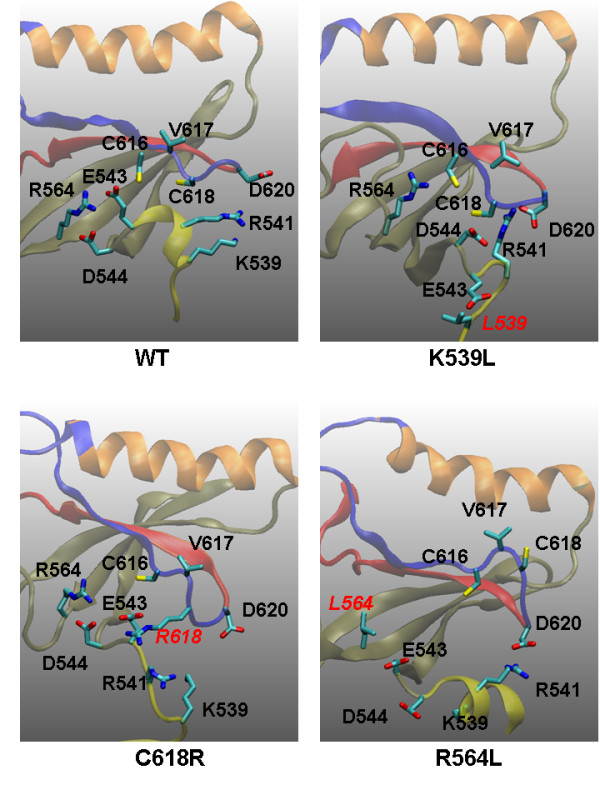
**Possible origins of mutational effects of K539L, C618R, and R564L derived from the snapshots of various mutant simulations at 44 ns**. The JH2 C-helix is shown in orange, the β-4 sheet in blue, and the β-5 sheet in red. In all three cases, the local conformation near V617 is distorted, although through different mechanisms. In K539L, L539 moves away from the highly-charged region of D620 and R541 and brings down E543, D544. In R564L, the strong interactions between R564 and E543/D544 are totally missing. In C618R, R618 moves toward E543/D544 and forms strong interactions with them.

In the simulation-derived structure (Figure 1), V617 is positioned to contact the JH1 activation loop, a result of the fold of the JH2 domain. A mutation altering the JH2 fold will likely affect the V617 position and hence the JH1/JH2 interface. V617 is located at the joint of the β-4 and β-5 sheets (Figure 7). These two beta sheets are connected through the hydrogen bonding network and anchored by surrounding residues. Figure 7 shows parts of those surrounding residues. A loop from K539 to D544 forms few interactions with the β-4 sheet: D620 is in a highly charged environment formed by K539 and R541. C616 and C618, through some water bridges, point to the charged environment formed by R564, E543, and D544. Simulation results indicate that mutations of the relevant residues significantly disrupt the local conformation for the following reasons (Figure 7):

***In K539L***, L539 moves away from the highly-charged region of D620 and R541 and moves E543 and D544 downward.

***In R564L***, the strong salt bridge interactions between R564 and E543/D544 are completely lost.

***In C618R***, R618 moves toward E543/D544 and forms strong salt bridges with them, disrupting the interaction between R564 and E543.

These conformational changes are consistent with the analysis of the JH1/JH2 interface (Figure 1, Figure 3, and Table 1). The results also suggest that the loop of residues 539 to 544 is important to support the local conformation near V617.

### L579F, L624P, and H606Q

**Figure 8:**
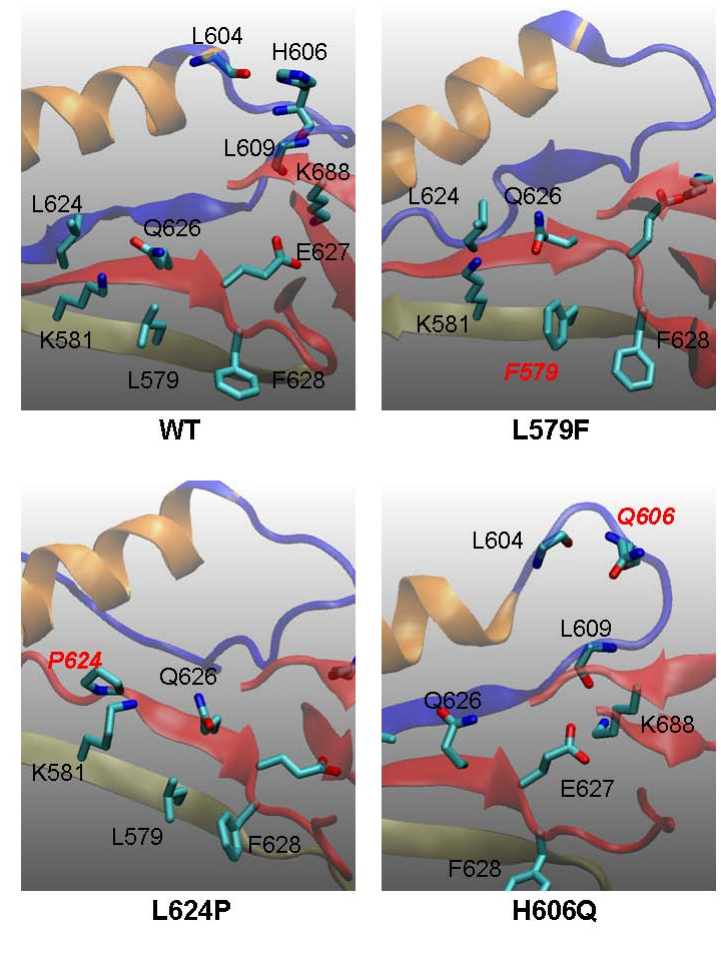
**Possible origins of mutational effects of L579F, L624P, and H606Q derived from the snapshots of various mutant simulations at 44 ns**. The JH2 C-helix is shown in orange, the β-4 sheet in blue, and the β-5 sheet in red. In L579F, F579 interacts with nearby F628, changes the surrounding packing pattern, and causes conformational changes between the β-4 and β-5 sheets. In L624P, P624 enforces a conformational change of the β-5 sheet. In H606Q, the interactions between the H606 side chain and the L604/L609 backbones are disrupted and the conformation of the loop (604 to 609) is changed.

Similarly, L624 is located in the β-5 sheet, L579 is located in a nearby beta-sheet, and H606Q is in the loop connecting the JH2 C-helix and β-4 sheet (Figure 8). All three mutants cause conformational changes in this region. In L579F, F579 interacts with nearby F628, changes the surrounding packing pattern, and causes the conformational changes between the β-4 and β-5 sheets. In L624P, P624 enforces a conformational change of the β-5 sheet because of the rigid conformation of proline. L579F and L624P cause local conformational changes but their mutational effects are relatively indirect and not as deleterious, consistent with the simulation results (Figure 3, and Figure 4).

H606Q disrupts the interactions between the residue 606 sidechain and the L604/L609 backbones, affecting the conformation of the loop (604 to 609) connecting the JH2 C-helix and the β-4 sheet and thus changing the position of V617 relative to the C-helix. This finding is consistent with the simulation results in that the I-2 and I-3 interactions are damaged severely (Figure 4) when the I-1 is moderately distorted (Figure 3). In this region, E627 and K688 form strong salt bridge interactions to maintain the local conformation. Mutations of these two positions or nearby residues may also result in strong deleterious effects.

### 14-del

In this mutant the entire exon-14 coding region is deleted (residues 593 to 622). Almost every residue of the JH1/JH2 interface is missing, except S591 and E952. Simulation results (Figure 3 and Figure 4) suggest that this mutant will very likely cause JAK2 constitutive activation.

### H587N and K603Q/N667K Double-Mutant

**Figure 9:**
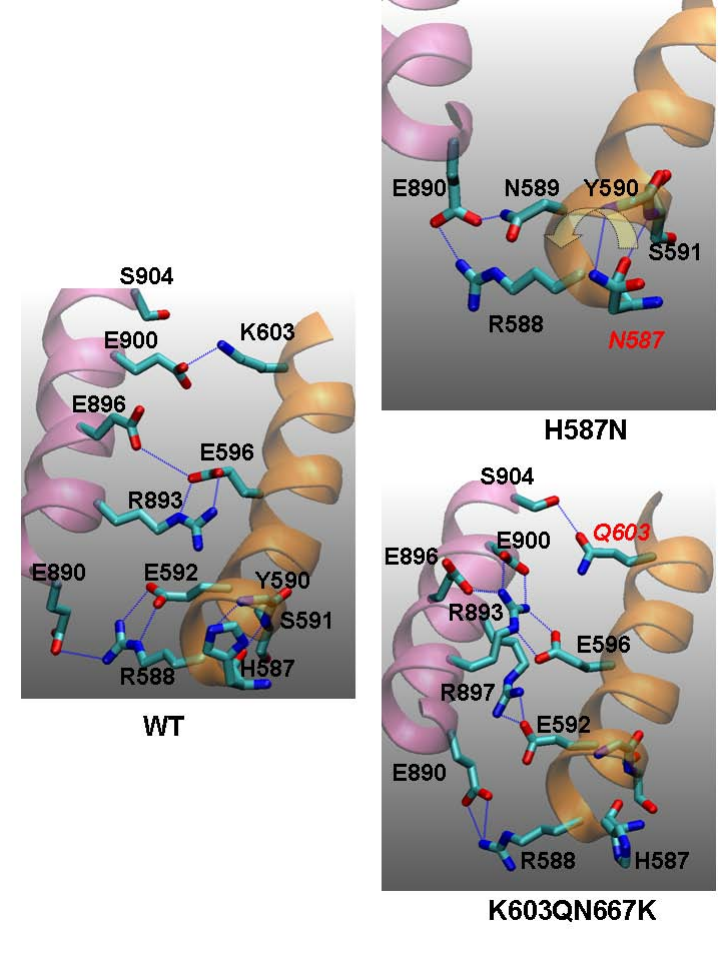
**Possible origins of mutational effects of H587N and the double mutant K603Q/N667K, derived from the snapshots of various mutant simulations at 44 ns**. The JH2 C helix is shown in orange and the JH1 C-helix is shown in pink. The H587 side chain forms hydrogen bonds with the Y590 backbone and the S591 backbone. In N587, the hydrogen bond network is modified. The S591 backbone now forms a hydrogen bond with the N587 backbone. The changes in hydrogen bond pattern result in a rotation of the JH2 C-helix (indicated by the yellow) as well as changes of the pattern of the hydrogen bond network between E890, R588, and N589. In K603Q/N667K, a control mutant, the JH1/JH2 interface seems to be maintained but the hydrogen bond network is significantly different from that of the wild-type.

Because H587 is exposed to solvent and H587N would constitute a polar-to-polar conserved mutation, this variation might not be expected to cause a significant mutational effect. However, simulation results indicate that H587N modifies hydrogen bond pattern of the 587 residue. The sidechain of H587 normally forms hydrogen bonds with the backbones of Y590 and S591; in the N587 variant, the S591 backbone forms hydrogen bonds with the backbone of N587 (Figure 9). The altered hydrogen bond pattern causes rotation of the JH2 C-helix and additional changes in the pattern of the hydrogen bond network between E890, R588, and N589. In N587, E890 now forms a new hydrogen bond with E589 and brings itself closer to the JH2 C-helix. The result is the disruption of the I-1 interactions, indicating that H587N is a deleterious mutation.

As mentioned above, the K603Q/N667K double-mutant exists in the JAK2s of other mammals, including pig, chicken, mouse, and rat; only human and pony sequences show the "un-mutated" sequences (K603/N667). Not surprisingly, the simulation results suggest that K603/N667 will be a benign mutation (Figure 3, Figure 4, and Table 1). However, the detailed I-1 interaction patterns are significantly different from those of wild-type JAK2 (Figure 9). Wild-type has the following hydrogen bonding or salt bridge pairs: K603/E900, E596/E896, E596/R893, E592/R588, and R588/E890. In K603Q/N667K, there is no salt bridge between Q603 and E900, since Q603 lacks a positive charge; Q603 forms a hydrogen bond with S904 instead, causing significant rearrangements of the interactions. Hydrogen bonding or salt bridge pairs in K603/QN667K JAK2 include Q603/S904, E596/R893, E896/R893, E900/R893, E592/R897, and R588/E890. The interactions in K603Q/N667K JAK2 seem stronger than in wild-type. Indeed, the fluctuation (standard deviation) of the torsion angle between JH1/JH2 C-helices is much smaller in K603Q/N667K than in wild-type (Figure 3). This finding suggests that K603Q/N667K could have more stable I-1 interactions and may imply that mutational studies based on murine models could lead to different results, especially when considering the effect on the I-1 interactions. For example, H587 is no longer held by the side chains of Y590 and S591 in K603Q/N667K, which may imply that a mutation at the 587 position, such as H587N, would have different effect in the K603Q/N667K mutant than in wild-type JAK2.

## Conclusion

In summary, we report a set of molecular dynamics simulations on clinically observed mutants along with control mutants. The results, which could not be obtained from simple homology-based models or other static structures, are consistent with available clinical and experimental evidence. Although the JH1/JH2 interface is derived from simulations and should be considered as putative, the anti-symmetrical JH1/JH2 interface seems very reasonable and we are able to explain the mutational effect for each mutant at atomic levels, including the presumably benign mutants V617C and K603Q/N667K. The results and analysis provide likely structural explanations of mutational mechanisms and may advance the understanding of JAK2 auto-regulation. It is clear that our simulation-derived structures are capable of reproducing all available experimental/clinical mutational data. However, it is important to emphasize that such success does not prove that these structures are correct before experimental structures are available.

Our simulation findings together with recent *JAK2 *mutation profiling results [34] suggest that molecular testing of JAK2 mutations should not be restricted to V617F and exon 12 mutations [43-45], but perhaps should extend to most of the pseudo-kinase domain coding region as well, especially the relevant residues shown in our atomic-level analysis.

## Methods

### Starting Structure

The only currently available theoretical structure model of the entire JAK2 protein, built using homology modeling methods, and co-evolution data [23,40], were used as the starting point. The original papers of this model have been cited over 30 times and have been extensively used to rationalize experimental/clinical evidence. Although it is relatively old, it is widely accepted in the JAK2 community[35], and has been utilized to explain structural effects [36,46], and has even been used as the starting model for other system (JAK1)[47]. The domain arrangement of this model is the current consensus accepted by those who are working on JAK2 crystal structures [48]. The inter-domain interaction between JH1 and JH2 was further verified by the DComplex server  and the reported binding free energy[49] between JH1 (residues 840 to 1129) and JH2 (residues 543 to 839) is -11.21 Kcal/mol for this homology model; while the value for the MD-simulation structure at 44 ns snapshot (WT) is -12.84 Kcal/mol. Both show that the JH1/JH2 inter-domain arrangement is reasonable. Based on the above reasons, we chose this model as our starting structure for all subsequent simulations.

### Homology Modelling for 14-del

The MODELLER software package (version 9v5) [50] was used to build the homology model for the 14-del variant; The sequences of 14-del JAK2 (deletion of residues S593 to N622) and wild-type JAK2 proteins were aligned using the ClustalW web server [51], resulting in a 30-residue gap between residue 592 and 623 (WT numbering). The aligned WT and 14-del sequences were used as the input alignment for MODELLER, and the WT structure mentioned above as the template structure. The modeller.automodel module in MODELLER with default parameters was used to build one model without any additional restraints. This MODELLER-generated 14-del structure then was used as the starting structure for the subsequent MD simulation of the 14-del variant.

### Molecular Dynamics Simulations

The homology-based model of the whole JAK2 assembly [40] was used as the initial structure for the wild-type and all mutant simulations, except for the 14-del mutant as described in the previous section. The following preparation steps were performed prior to molecular dynamics simulations, using the VMD software package (version 1.8.6, )[52]. All ionizable residues were considered in the standard ionization state at neutral pH condition. The whole protein then was put in a water box, filled with TIP3P water[53], with at least 15 Å buffer distance in any direction, resulting in a roughly 130 Å × 120 Å × 90 Å box containing JAK2 and water molecules. Sodium and chloride ions were added randomly in space (but were kept initially at least 4.7 Å away from any solute atoms) to reach the physiological concentration of sodium ions (0.14 M) and overall electric neutrality. The complex was oriented to align its longest axis with the x-axis of the water box, and its center of mass was placed at the center of the box. Water molecules less than 1.4 Å from any atom of the complex were removed. The resulting system contained 138,824 atoms for the wild-type (JAK2:17,657; water: 121,062; sodium ion: 53; chloride ion: 52) and 130,007 atoms for the 14-del mutant (JAK2:17,209; water: 112,698; sodium ion: 49; chloride ion: 49).

All molecular dynamics (MD) simulations were performed using the NAMD package (version 2.6) [54] with the CHARMM27 force field [55,56]. Default parameters and settings were used except as mentioned below. Periodic boundary conditions were used along with the isothermal-isobaric ensemble (*NPT*) at 1 atm and 298 K using an extended system pressure algorithm [57] with effective mass of 500.0 *amu *and Nosé-Hoover thermostat[58,59] with effective mass of 1000.0 kcal/mol-ps^2^. The smooth particle mesh Ewald (PME) method [60] was employed. A B-spline interpolation order of 4 was used, combined with 120, 120, and 75 FFT grid points for the lattice directions x, y, and z, respectively. Non-bonded interactions were treated using an atom-based cutoff of 12 Å with switching van der Waals potential beginning at 10 Å. Numerical integration was performed using the leap-frog Verlet algorithm with 1 fs time step. Covalent bond lengths involving hydrogen were constrained using the SHAKE algorithm [61,62].

The following procedure was used prior to the data collection for the wild-type and 14-del mutant. All atoms of the protein complex were first restrained at their starting structure positions with a force constant of 50 kcal/mol/Å^2^. Water and ion molecules were first energy-optimized and then underwent the following simulation annealing to ensure the proper equilibration. The temperature was increased from 0 K to 600 K at the rate of 1 K per ps and maintained at 600 K for 500 ps. Then the temperature was decreased to 300 K at the rate of 1 K per ps and maintained at 300 K for 500 ps. The system then was kept at 300 K for 4 ns. The total equilibration time was about 5 ns in this step. The system was next brought down to 0 K and the same 5 ns equilibration was repeated, resulting in an equilibration simulation of total 10 ns for water molecules and ions. The water/ion-equilibrated systems, both of the wild-type and 14-del, then were used as the starting point for the production simulations. All mutants, except 14-del, were created from the water/ion-equilibrated wild-type structure through the VMD package. For all simulations, the restraint force constant then was gradually reduced to 3 kcal/mole/Å^2 ^during a 500-ps period, followed by 2,000-step energy-minimization for all atoms and a 300-ps heating period with the temperature increased from 0 to 300 K at the rate of 1 K per ps. Production MD simulations of 60 ns were then performed for all simulations without any constraint or restraint. Trajectories were saved at the frequency of 1 ps.

### Simulation Stability

**Figure 10:**
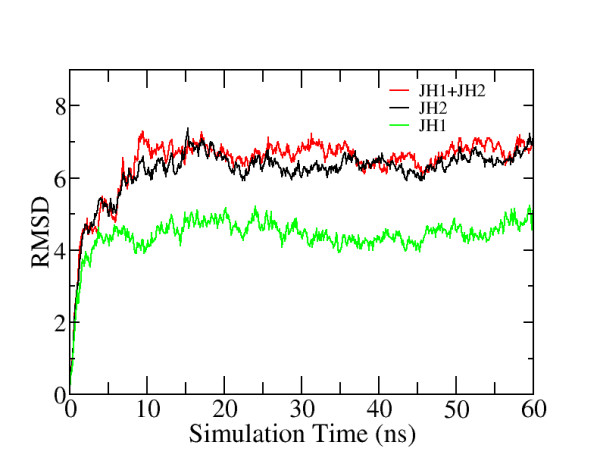
**Heavy atom root-mean-squared deviation (RMSD, in Å, relative to the initial structure) of JH1 domain (defined as residue 840 to 1129), JH2 domain (defined as residue 543 to 839), and of JH1/JH2 together (defined as residue 543 to 1129) for the wild-type JAK2**.

Figure 10 shows the heavy atom root-mean-squared deviation (RMSD, in Å, relative to the initial strucuture) of JH1 domain (defined as residue 840 to 1129), JH2 domain (defined as residue 543 to 839), and of JH1/JH2 together (defined as residue 543 to 1129) for the wild-type JAK2. RMSD is commonly used as an indicator of stability of MD simulations. The RMSD curves of individual JH1 and JH2 domains indicate simulations of both domains are stable after ~20 ns. The RMSD curve of combined JH1/JH2 indicates that the relative positions of JH1/JH2 as well as their tertiary interactions are also stable after ~20 ns. Simulations of other mutants exhibit similar behaviours. Those results suggest that the simulations reported here are converged and the corresponding derived analysis is reliable in the timescale simulated.

## Authors' contributions

TL designed and performed all calculations and data analysis, and wrote the first draft. WM provided clinical and experimental data, and modified the draft. XZ provided clinical and experimental data, and modified the draft. HK provided clinical and experimental data, and modified the draft. MA initiated the project, coordinated efforts between co-authors, and modified the draft. All authors read and approved the final manuscript.
